# Valence as principal dimension of the semantic space in primary progressive aphasia semantic variant

**DOI:** 10.1093/braincomms/fcaf281

**Published:** 2025-07-23

**Authors:** Antonietta Gabriella Liuzzi, Karen Meersmans, Kevin Statz, Nathalie Dusart, Jolien Schaeverbeke, Yolande A L Pijnenburg, Simon De Deyne, Gerrit Storms, Rik Vandenberghe

**Affiliations:** Laboratory for Cognitive Neurology, Department of Neurosciences, Leuven Brain Institute, KU Leuven, Leuven 3000, Belgium; Laboratory for Cognitive Neurology, Department of Neurosciences, Leuven Brain Institute, KU Leuven, Leuven 3000, Belgium; Laboratory for Cognitive Neurology, Department of Neurosciences, Leuven Brain Institute, KU Leuven, Leuven 3000, Belgium; Neurology Service, University Hospitals Leuven, Leuven 3000, Belgium; Laboratory for Cognitive Neurology, Department of Neurosciences, Leuven Brain Institute, KU Leuven, Leuven 3000, Belgium; Laboratory for Cognitive Neurology, Department of Neurosciences, Leuven Brain Institute, KU Leuven, Leuven 3000, Belgium; Alzheimer Centrum, Amsterdam University Medical Centre, Amsterdam 1081, Netherlands; Melbourne School of Psychological Sciences, Melbourne University, Melbourne 3010, Australia; Laboratory for Experimental Psychology, KU Leuven, Leuven 3000, Belgium; Laboratory for Cognitive Neurology, Department of Neurosciences, Leuven Brain Institute, KU Leuven, Leuven 3000, Belgium; Neurology Service, University Hospitals Leuven, Leuven 3000, Belgium

**Keywords:** semantic variant, primary progressive aphasia, valence, semantic space

## Abstract

Previous studies of the semantic variant of primary progressive aphasia have determined which word features render single words more vulnerable. Here, we applied a graph-based approach and modelled the pathological changes at the network level. In healthy individuals, the two principal dimensions for the organization of the word meaning network are concreteness and valence. Ten semantic variant of primary progressive aphasia cases (mean age 66 years, 6 female, Clinical Dementia Rating: 3 patients = 0; 6 patients = 0.5; 1 patient = 1) participated as well as 79 matched controls. A set of 32 words were selected a priori to cover a broad range of valence and concreteness. In a triad judgement task (total of 192 trials), three words were presented on the screen at the corners of a triangle. Participants had to select which word pair was most closely associated in meaning. Responses were analysed using multidimensional Bayesian scaling. In controls, the first principal dimension was determined by word concreteness (β = 0.39, *P* = 0.013) and valence (β = 0.34, *P* = 0.028) and the second by word dominance (β = 2.09, *P* = 0.0009) and arousal (β = −1.45, *P* = 0.009). In patients, the semantic network changed to a circumplex configuration with valence as the unique dimension (dimension 1: β = −0.72, *P* = 0.0005; dimension 2: β = 0.46, *P* = 0.006) and no effect of word concreteness. In the semantic variant of primary progressive aphasia, there is relative preservation of word valence as one of the organizational principles for the word network despite the loss of concreteness as organizing principle.

## Teaser

Liuzzi *et al*., applied a graph-based approach on a triad judgement task in 10 semantic variant of primary progressive aphasia (SV PPA) and 79 controls. In SV PPA, the word meaning network shifts from concreteness and valence-based organization to a circumplex configuration with valence as a single organization principle.

## Graphical Abstract

**Graphical Abstract:**
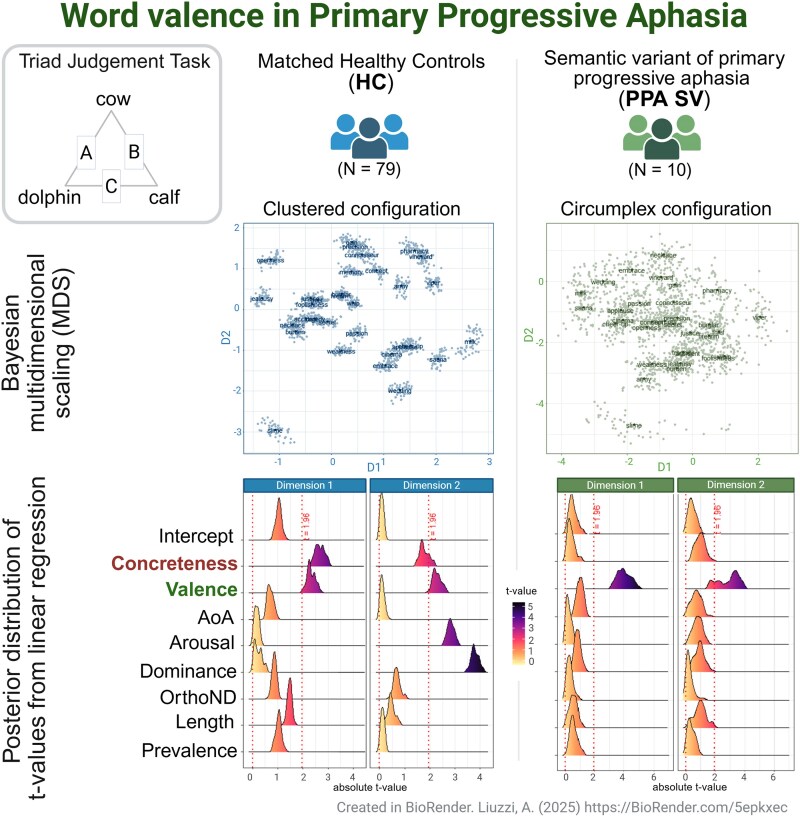

## Introduction

The semantic variant of primary progressive aphasia (PPA SV) is associated with a gradual loss of word meaning and semantic knowledge. This manifests itself first in word finding difficulties and subsequently in word comprehension loss, typically first for subordinate categories^1^ such as specific food ingredients. As the disease progresses, object identification is also affected. For instance, participants may no longer recognize certain ingredients on their plate. The combined word production and comprehension loss and object recognition deficit has led to the hypothesis of a amodal, or, alternatively, multi-modal semantic deficit. PPA SV has been highly influential in the development of current theories about the representation of word meaning and concepts in the human brain, such as the heteromodal theory^2^ and the hub-and-spoke model.^3^

In the neurotypical population, it has been established for decades that two of the main dimensions along which the semantic space is organized are concreteness and word valence.^4^ Much progress has been made since in modelling the semantic system from a network perspective. A key challenge remains to model the semantic network at the individual level rather than based on group data. Individually based modelling is necessary for clinical applications given the inter-individual heterogeneity in the patient groups.

In PPA SV, both for comprehension and production, the effect of concreteness has been extensively studied. Whereas, in case of a neurological dissociation between abstract and concrete words, abstract words are most often affected more than concrete words, the reverse pattern has been described in PPA SV.^5-7^ Such a concreteness reversal effect has been reported based on scene description,^5^ triad judgement task with abstract and concrete verbs,^6^ the autobiographical memory interview^8^ and other tasks. The concreteness reversal effect is not systematically present in all patients^8,9^ nor for all abstract categories.^7^ The reduced concreteness of nouns in spontaneous speech correlates with anterior inferior temporal and parahippocampal cortical volume.^5^ This pattern is opposite to what is seen in behavioural variant of frontotemporal dementia cases with inferior and medial frontal damage^5^ and to most patients with aphasia from other aetiologies. The concreteness reversal has been attributed to degraded visual knowledge^5^ or to a preference for words that can be used in a broader range of linguistic contexts (words with a higher semantic diversity).^8^ The latter hypothesis is in line with a multidimensional view on word abstractness.^10^

The second main dimension of the neurotypical semantic network, word valence, has been much less investigated in this patient group, despite its key role as an organizing principle of the semantic space in the intact brain. Concept and emotion processing are entwined in the human brain.^11^ Language researchers have identified emotion as an important mechanism to ground (abstract) concepts in bodily experience,^12^ while emotion researchers have put forward the idea that words function as unifying labels for (un)pleasant and/or arousing experiences (e.g. identifying separate instances of an increase in heart rate, a flushed face, etc. as *anger*).^13,14^ Previous studies have shown that knowledge of emotions is impaired in PPA SV.^15,16^ Comprehension of emotional concepts was significantly correlated with facial emotion recognition in PPA SV, but not controls. Further research shows that the perception of valence in facial expressions (i.e. pleasant or unpleasant) is preserved in PPA SV, but not the identification of discrete emotions (e.g. anger and happiness).^17^ Studies of the effect of word valence on semantic processing in neurodegenerative syndromes are rather scarce, as most emotion research focusses on non-word stimuli. A study in eight PPA SV cases^18^ revealed that for a given word, recognition of its valence related to performance on lexical decision for that word but not to performance on associative-semantic comparison. This may indicate that word valence is already processed at an early single word recognition^19^ stage, rather than the associative-semantic processing level, in line with electrophysiological^20^ and affective priming evidence.

A network approach akin to what psycholinguistics have been using has only rarely been applied in PPA SV. One main reason may be the difficulty of modelling the semantic network reliably at the individual level. Recently, experimental psychology has made important steps forward in its capability to map the semantic space,^21^ for instance based on Bayesian multidimensional scaling based on triadic comparisons.^22^ These methods have been applied to study semantic processing in Alzheimer disease,^23^ with some methodological caveats.^24,25^

The tasks we used to collect data suitable for individual-basis semantic mapping were adopted from experimental psychology and included a triad judgement task^18,23,24,26^ and a pairwise judgement task.^27^ Pairwise judgement involves forced-choice discrimination between two nouns along a pre-specified dimension, in the current study concreteness and valence. In the triad judgement task three nouns are presented along an imaginary triangle and participants have to indicate which edge is closest in meaning. The task set-up has two characteristic features. The semantic space was sampled comprehensively based on a fixed and limited set of words without presenting each possible pair or triad, as that would be result in excessive length of the test. This subsampling of the possible triads requires judicious selection of the triads. A second key feature of the task is that the triads could consist of very dissimilar words. In the triad task, pairs with weak similarity are also informative for modelling the semantic network.^26^

We modelled the semantic space at the individual level to evaluate in which manner PPA SV affects its organization. The primary interest was to determine whether the principal dimensions of concreteness and valence were differentially affected. Other classical word features were also included in the analyses, including, among others, arousal and dominance.^4-30^ We also determined whether the semantic space was more randomly organized or, to the contrary, in an overly structured and stereotyped manner.

## Methods

### Participants

PPA SV patients were recruited via UZ Leuven Memory Clinic and classified according to international recommendations for diagnosis.^31^ Given the demands of the experimental tasks, only patients in early stages of the disease were included. The PPA SV patients (*n* = 10; 6 women and 4 men) had a mean age of 66 (SD = 5.63) and mean education of 14.7 years (SD = 2.00).

Healthy control (HC) subjects were recruited from the Flemish Prevent Alzheimer’s Disease Cohort KU Leuven (F-PACK) longitudinal observational cohort.^32^ In addition, nine participants who expressed interest in the study but who did not belong to the F-PACK cohort were included (e.g. spouses). These participants were only included in the analysis of the behavioural performance on the semantic test battery. Participants in the F-PACK study were recruited from the population via advertisements in newspapers and magazines. Inclusion criteria were age between 65 and 80 years old, a mini mental state examination score ≥27, a clinical dementia rating global score of zero and scores on a standard neuropsychological examination within published norms. The exclusion criteria were a significant neurological or psychiatric history, focal brain lesions based on MRI, a history of cancer, a counter indication for MRI (pacemaker, metal implants or severe claustrophobia) or exposure to radiation for research procedures within the year prior to the [18F]-flutemetamol amyloid-PET scan. The subgroup included in this study (*n* = 79; 41 women and 38 men) had a mean age of 71.97 (SD = 6.33) and mean education of 14.83 years (SD = 3.05) (Table 1).

**Table 1:** Group averaged demographic and neuropsychological characteristics

	PPA SV	Controls	*P*-value
**Participants, *n***	*n* = 10	*n* = 79	
**Age, years**	66 ± 5.63 [58, 76]	71.97 ± 6.33 [58, 83]	
**Sex, male/female**	4 M/6F	38 M/41 F	
**Education, years**	14.70 ± 2.00 [12, 17]	14.89 ± 3.20 [10, 23.5]	
**AVLT total learning (/75)**	30.30 ± 12.16 [7,52]	48.31 ± 9.84 [26, 69]	t(10) = −4.46, *P* = 0.001
**AVLT delayed recall, %**	0.46 ± 0.35 [0, 0.83]	0.83 ± 0.17 [0.43, 1.15]	*W* = 0, *P* < 0.001
**AVLT** d**elayed** r**ecall (/15)**	9.80 ± 3.99 [0, 15]	12.92 ± 2.04 [6, 15]	*W* = 192.5, *P* = 0.0035
**Boston naming test, %**	39.75 ± 27.50 [5, 93.33]	55.35 ± 3.71 [44, 60]	*W* = 134, *P* < 0.001
**Animal verbal fluency 1 min**	12.8 ± 7.55 [5, 24]	19.97 ± 5.74 [11, 43]	*W* = 219, *P* = 0.012
**Pyramids and palm trees test (/52)**	44.9 ± 5.84 [36, 52]	NA	
**Semantic association test PALPA**			
** PALPA 9 (/160)**	147.6 ± 14.897.77 [116, 160]	NA	
**PALPA 45 (/40)**	35.2 ± 5.22 [25, 40]	NA	
**PALPA 49 (/30)**	21.6 ± 6.92 [9, 28]	27.43 ± 1.70 [22, 30]	*W* = 173.5, *P* = 0.001
**Werkwoorden-en zinnentest (/120)**	105.7 ± 10.251.34 [86, 116]	NA	
**DIAS**			
**Consonant**	14.8 ± 0.65 [13,15]		
**Vocal**	15 ± 0 [15,15]		
**Correct** s**equential**	6 ± 0 [6,6]	NA	
**Correct** a**lternating**	5.9 ± 0.32 [5,6]		

AVLT, auditory verbal learning test; PALPA9 word and pseudoword repetition; PALPA 45 lexical decision; PALPA 49 words semantic association test for high and low imageable words. DIAS = Diagnostisch Instrument voor Apraxie van de Spraak (English translation: Diagnostic Instrument for Apraxia of Speech). Werkwoorden en zinnentest (English translation: Verbs and sentences test). W = Wilcoxon rank-sum test.

### Neuropsychological evaluation

Within the F-PACK study, participants undergo 2-yearly neuropsychological evaluation over a period of 10 years. This protocol consists of Mini Mental State Examination (MMSE), Clinical Dementia Rating (CDR), Auditory Verbal Learning Test (AVLT), Raven’s Progressive Matrices (RPM), Animal Verbal Fluency (AVF) and Phonological Verbal Fluency (PVF), Boston Naming Test (BNT), PALPA-49, Trial Making Test (TMT), Buschke Selective Reminding Test (BSRT). We report the mean performance based on the most recent neuropsychological evaluation. The patient group also underwent neuropsychological evaluation, including tests of general neurocognitive functioning (CDR, MMSE), memory (AVLT), linguistic ability (BNT, AVF, PALPA49, PALPA45, PALPA9, Birmingham Object Recognition Battery, Pyramids and Palm Trees, Werkwoorden-en Zinnentest, Diagnostic Instrument for Apraxia of Speech), and non-verbal fluid intelligence and reasoning (Raven’s coloured progressive matrices (CDR)) (Table 1).

### Neuroimaging protocol

Structural and functional images were acquired on a 3T Philips Achieve system with 32-channel head coil. Structural images were acquired using a T1-weighted 3D turbo-field-echo sequence (voxel size = 1.05 × 1.05 × 1.2 mm^3^, 170 slices, repetition time = 9.7 ms). Functional images were acquired using a T2* sequence for a duration of 8.3 min (repetition time =2 s, voxel size = 3 × 3 × 3 mm^3^, FOV = 224 × 131.8 × 224, multiband factor = 2, number of slices = 60). Subject was instructed to keep their eyes open during resting state data acquisition and focus on a fixation cross while thinking of nothing in particular.

### Cortical thickness processing

**Figure 1:**
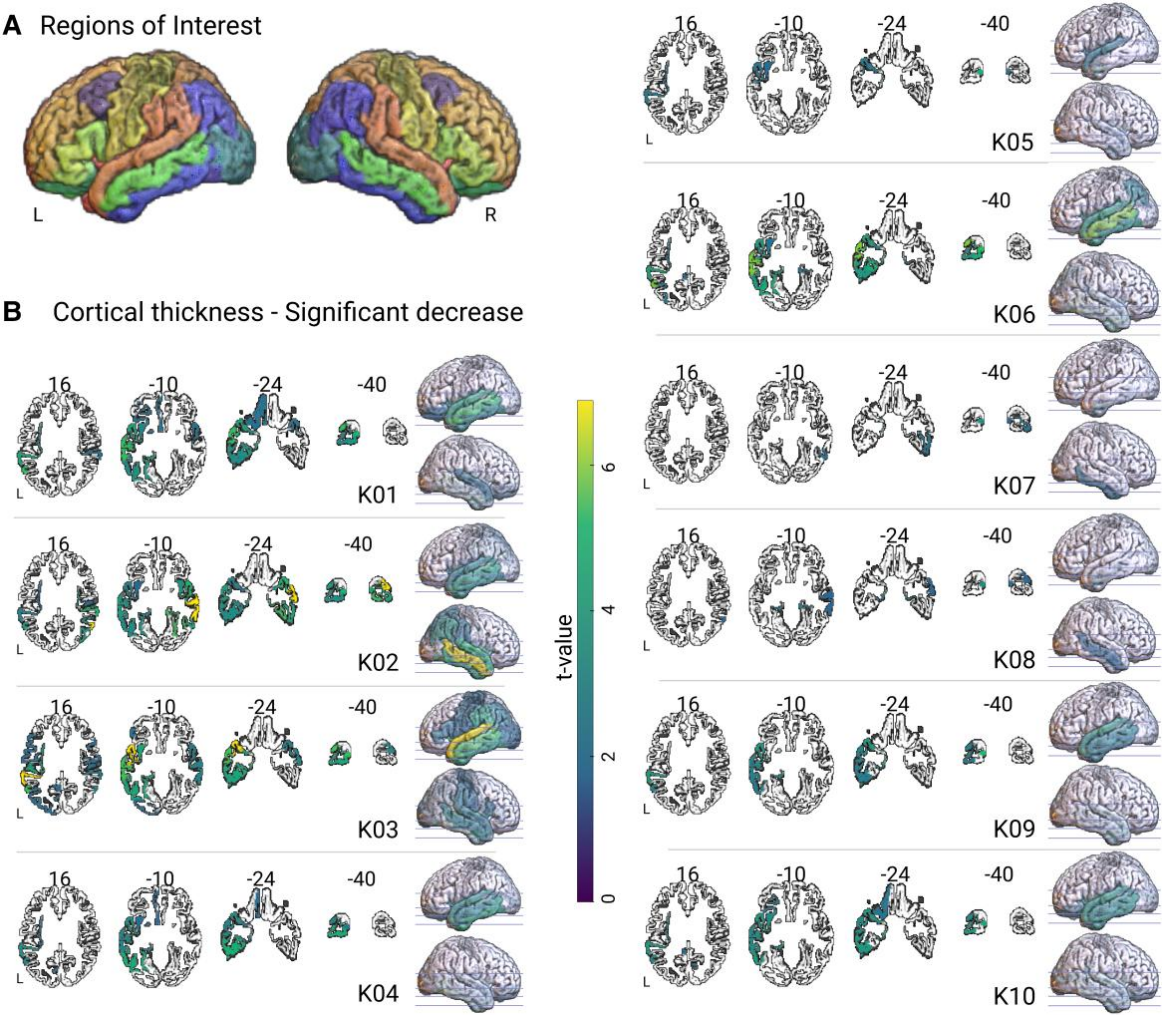
**Cortical thickness**. **(A)** For each hemisphere, rendering of the 34 ROIs derived by the Desikan–Killiany atlas. **(B)** For each patient, axial slices and rendering showing the significant (*P* < 0.05) decrease (minus *t*-value) of the cortical thickness obtained by using a Crawford and Howell’s modified *t*-test. The analysis is region-based.

For each of the 34 regions of interest (ROIs) per hemisphere defined by the Desikan–Killiany atlas (Fig. 1A),^33^ average cortical thickness measurements were obtained from T1-weighted 3T MRI scans using the *recon-all* processing pipeline provided in FreeSurfer^34^ (version 7.4.1). This pipeline estimates cortical thickness by calculating the mean distance between vertices of an estimated grey–white matter boundary surface and a pial surface.^35^ Regional average thicknesses were extracted directly from the *aparc.stats* and *aseg.stats* output files generated by FreeSurfer. All cortical parcellation and segmentation outputs were visually inspected for errors, which did not occur in this dataset. Consequently, no manual corrections were applied.

### Experimental procedures

#### Stimulus set

The same set of stimuli was used for the two tasks. Thirty-two words were selected from the Dutch Small World of Words dataset^36^ along the concreteness^37^ and valence dimensions,^38^ controlled for arousal, frequency,^39^ orthographic neighbourhood density,^40^ prevalence^41^ and word length and matched as good as was possible on age of acquisition^37^ and dominance^29^ (Table 2; Supplementary Fig. 1 and Supplementary Table 1). Words with concreteness ratings between 1.5 and 2.5 were considered abstract and words with concreteness ratings between 4 and 5 were considered concrete (on a 1–5 scale).^37^ Words in the middle of scale were excluded due to typically high variability in concreteness ratings in this range.^42^ Words with valence between 2 and 4 were considered negative and words with valence between 4 and 6 were considered positive (on a 1–7 rating scale). In addition, low-frequency words (log10 frequency < 1) and low-prevalent words (known by <98% of the general population) were excluded.

**Table 2:** Characteristics of the four classes of words used in the two tasks (mean and SD)

	Abstract positive	Abstract negative	Concrete positive	Concrete negative	Anova *P*-value
**Concreteness**	2.04 ± 0.3	2.03 ± 0.3	4.4 ± 0.3	4.4 ± 0.3	<10^−15^
**Valence**	5.2 ± 0.4	2.6 ± 0.4	5.3 ± 0.4	2.8 ± 0.4	<10^−15^
**AoA**	9.5 ± 1.0	8.6 ± 2.0	7.7 ± 2.0	7.5 ± 1.1	0.06
**Arousal**	4.5 ± 0.6	4.4 ± 0.7	4.2 ± 0.7	4.8 ± 0.8	0.34
**Dominance**	4.7 ± 0.3	4.0 ± 0.8	4.3 ± 0.4	4.6 ± 0.7	0.06
**OrthoND**	1.8 ± 2.3	3.0 ± 5.5	0.8 ± 1.8	4.0 ± 3.1	0.28
**Length**	7 ± 1.2	7.0 ± 1.6	7.3 ± 1.9	6.1 ± 1.8	0.50
**Frequency**	2.9 ± 0.6	2.3 ± 0.6	2.4 ± 0.6	2.4 ± 0.7	0.32
**Prevalence**	0.99 ± 0.01	0.99 ± 0.01	0.99 ± 0.01	0.99 ± 0.01	0.33

AoA, age of acquisition; OrthoND, orthographic neighbourhood density.

#### Experiment design

The experimental tests were 3-fold: a pairwise valence judgement, a pairwise concreteness judgement and a triad judgement task.

The experimental tests were administered via an online platform for behavioural research (https://gorilla.sc). Controls completed the task at home, without supervision and at a time that was convenient for them. It was not necessary to complete all three tasks at once. Every task is self-paced and no time limit was set. Participants were invited via email and, after expressing interest in the study, participants received an instruction manual, informed consent form and personal code to login to the platform. The use of this code ensured anonymity and allowed the subject to complete the tasks whenever he or she wanted. To ensure data quality, we inspected the reading time of the instructions, the reaction times (very short reaction times suggesting that responses were given mindlessly) and task-specific measures of consistency. First, the participants performed the pairwise concreteness judgements, followed by the triad judgements and the pairwise valence judgement. Two HC participants, out of 79, did not perform all tasks. Accordingly, they were not included in the final sample size.

The patients were tested on-site using the same platform and set-up. They were tested on-site in order to be able to provide assistance with the conduct of the experiment more readily when needed.

Task-specific instructions were shown before the start of every task and participants could return to the instructions throughout the experiments.

##### Triad judgements

The triad task was modelled after De Deyne *et al*.^26^ Participants were presented with three words placed at the corners of an equilateral triangle. The edges were labelled A, B or C and participants were instructed to select the word pair that they considered to be most similar. The response options (A, B and C) were presented beneath the triads, each choice in a box, and participants were instructed to click on the box corresponding to the word pair they considered to be most similar. The stimuli were displayed in Open Sans font at a size of 14px.

A subset of 192 triads was selected from all possible combinations. When selecting triads, a trade-off between presenting as many combinations as possible and repeating word pairs in several triads was considered. When a pair is only presented once, the similarity estimates derived depend heavily on what the third word is (e.g. cow–dolphin–music versus cow–dolphin–calf). When a pair is presented in multiple triads, this effect is mitigated. First, triads were selected randomly ensuring that every word occurred at least 12 times. Next, combinations were selected from those that did not yet occur in the dataset in order the maximize the combinations that are presented. Finally, the triad set was further optimized by replacing triads that contain pairwise combinations that only occur once. In the final triad selection, only three pairwise combinations occur only once. A pair would be included in maximally six triads. The majority of word pairs were presented in two or three triads. Every individual stimulus word was presented 18 times. Twenty-four triads were repeated twice throughout the task to allow for examination of within-subject consistency. Trial sampling was the same for all participants and trial presentation was randomized across participants. Consistency will be expressed as the proportion of consistent responses within the 24 duplicate trials. The total duration of the triad judgement task was ∼30 min.

##### Pairwise judgements

The structure of the pairwise judgment tasks for concreteness and for valence was identical: pair of words was presented on the horizontal meridian, one word to each side of the screen. The instruction (i.e. the word *abstract* or *concrete* in the concreteness judgements task or the word *positive* or *negative* in the valence judgement task) appeared at the top of the screen. The stimuli were presented in Open Sans 400 font, size 25px, with the word pairs in white against a grey background, and the instruction word in light grey.

The concreteness and the valence judgement tasks were presented separately. In the pairwise concreteness judgement task, participants were instructed to select the more concrete or the more abstract word, depending on the instruction. In the pairwise valence judgement task, they were instructed to select the more positive or the more negative word for a pair of words, depending on the instruction. Participants were instructed to click with a cursor on the word of their choice. Participants were explicitly instructed to make a relative judgement. For instance, when two positive words are presented, they were asked to select the word that is more positive.

A total number of 256 word-pairs were presented. The same pairs were used in both the valence and concreteness judgement task. Word pairs were selected so that every word occurred 16 times. We selected 4 × 16 pairs from each quadrant in the valence × concreteness configuration (i.e. the words in the pair share both valence and concreteness), 4 × 32 pairs that have one dimension in common (e.g. pairs of positive words, pairs of concrete words, etc) and 2 × 32 pairs that share no dimensions (e.g. a negative concrete word with a positive abstract word). Every pair was repeated twice in every task with a different instruction, which allows for the assessment of consistency across responses. Word pairs were presented in 16 blocks that did not contain the same pair twice. Within each block, the same word occurred maximally five times. The presentation order of the blocks was randomized over participants. The total duration of each of the two pairwise judgement tasks was ∼30 min.

### Statistical analysis

#### Cortical thickness

The cortical thickness analysis using the Desikan–Killiany atlas^33^ revealed a clear left-hemisphere predominance of pathology in seven patients, with more right-hemisphere involvement in K02, K07 and K08. In K02 and K08, the most significant decrease of cortical thickness occurred in the right middle temporal gyrus, while in K07 the most significant decrease occurred in the right inferior temporal gyrus. It is worth noting that the analysis is parcel-based, meaning that it does not allow for investigation of intra-parcel variations in cortical thickness. For visualization (Fig. 1B), average thickness of each PPA SV patient was compared with the average thickness of a group of 64 control subjects using a Crawford and Howell’s modified *t*-test.

#### Data quality

We conducted a two-way ANOVA with group as between-subjects factor (two levels: SV versus controls) and task as within-subjects factor (three levels: pairwise valence judgement, pairwise concreteness judgement, triad judgement) and reaction times as outcome. Given the unbalance design, ANOVA with Type III sums of squares was adopted.

Consistency of responses was also evaluated. As mentioned above, within the concreteness and within the valence pairwise judgement tasks, every word pair was presented twice, with opposite instructions (select the abstract word in one of the two trials, the concrete word in the other trial; select the positive word in one of the two trials, the negative word in the other trial). For the concreteness and the valence pairwise judgement tasks, task-specific measures of consistency were calculated by computing the number of pairs with consistent responses across the two presentations/the number of total pairs.

**Figure 2:**
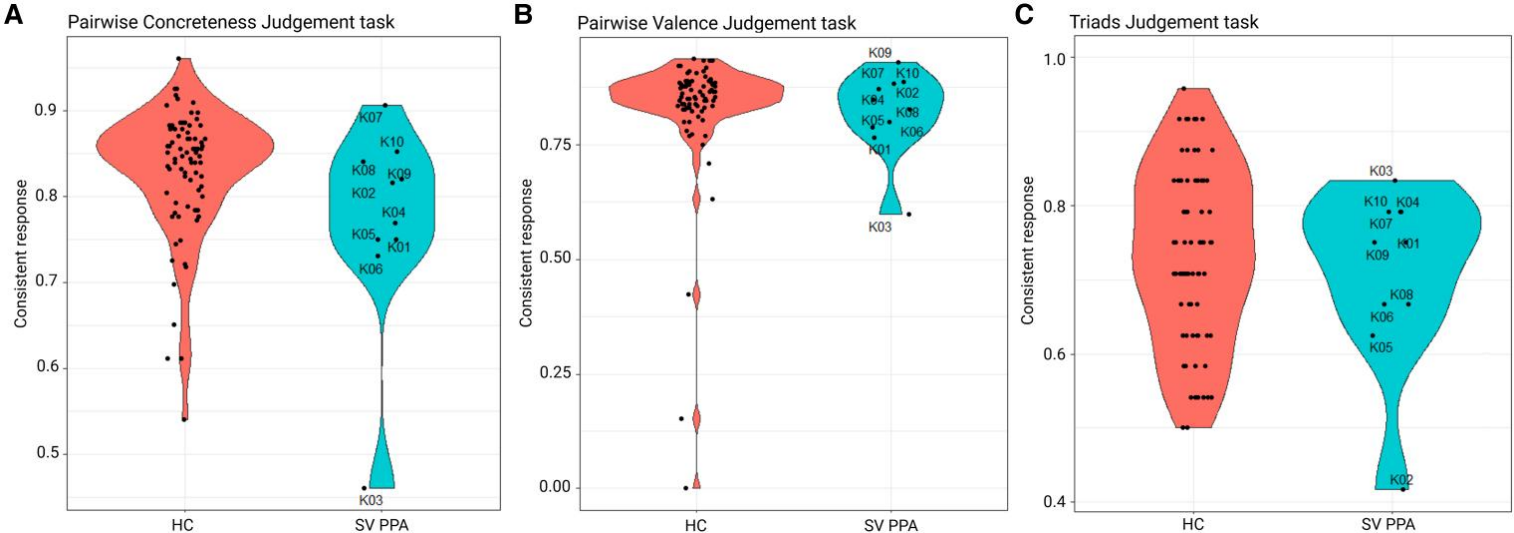
**Task-specific measures of consistency.** (A–C) Violin plots showing distributions of subject-specific consistency of response for HC and PPA SV for the **(A)** pairwise concreteness judgement task, **(B)** pairwise valence judgement task, and the **(C)** triad judgement task. For the concreteness and the valence pairwise judgement tasks, consistency was calculated by computing the number of pairs with consistent responses across the two presentations/the number of total pairs. For the triad judgement task, consistency was analysed by computing the number of repeated triads with the same response/number of repeated triads. HC, healthy control (*N* = 77); SV, semantic variant of PPA (*N* = 10).

For the triad judgemental task, as described above, 24 triads were presented twice. Consistency was analysed by computing the number of repeated triads with the same response/number of repeated triads (Fig. 2C).

#### Triad judgements

Triadic judgements were examined using a Bayesian approach to multidimensional scaling (MDS) especially developed for triadic comparisons.^22^ This approach uses graphical modelling to generate latent semantic 2D configurations from the triad responses. The main assumption is that triadic decisions are based on a shared semantic space and that the pairwise distances in this space determine the response. The underlying intuition of an odd-one-out decision is that a word is more likely to be chosen as odd-one-out if the remaining two words are more similar. The probability of choosing a word as the odd-one-out in a triad is thus proportional to the distance between the other two words. Importantly, this Bayesian approach to MDS formalizes individual differences by evaluating how the word chosen as odd-one-out follows the inferred underlying semantic representation of the stimuli. Accordingly, the intuition is that less impaired people will make the decision predicted by the semantic structure of the entities and more impaired people will deviate from those predictions because of an impaired (or lost) common semantic representations or common semantic dimension/s. In our experiment, participants were instructed to select the word pair that was most similar, effectively identifying the third word as the odd-one-out. The model assumes a uniform distribution on the latent coordinates, generating pairwise distances using the Minkowski metrics. The probability of a word being chosen as the odd-one-out is expressed proportionally to the pairwise distance between the other two words, as an exponential decay. The smaller the distance between A and B, the higher the probability of choosing C as the odd-one-out. Finally, Markov-Chain Monte–Carlo methods are used to sample coordinates from the joint posterior distribution dependent on the model and the data. The main advantage of this Bayesian approach is that the samples represent uncertainty on the coordinates. The greater the impairment, the higher the uncertainty about the locations of the stimuli, and the more the configurations will tend to resemble a circumplex configuration. Configurations were first obtained by pooling all data for HC and PPA SV, respectively. To interpret the resulting configuration, we used linear regression for the two dimensions separately with the coordinates as dependent variables and the stimulus ratings (prevalence, word length, orthographic neighbourhood density, dominance, arousal, age of acquisition, valence and concreteness) as predictors. Furthermore, a linear regression for each of the two dimensions was repeated on the 50 sample coordinates from the posterior distributions.

Lee *et al*.^22^ also propose a measure of spatial randomness that can be applied to the configuration to measure clustering. They demonstrate that spatial randomness increases with severity of cognitive impairment. Here, we applied this measure to quantify the change in semantic structure in patients. Spatial randomness *R* is defined as the ratio of the average nearest neighbour Minkowski distance in the generated configuration and the average neighbour distance in 10 000 random configurations. This metric is calculated for 50 samples from the posterior distribution. An *R*-value of 1 indicates complete spatial randomness (i.e. nearest neighbour distance in the observed and random configuration are equal). An *R*-value < 1 represents increasing levels of clusters, while R > 1 represents a shift towards a regular grid, which implies a spatially random MDS configuration lacking of semantic clustering.

#### Pairwise judgements

Based on the responses in the pairwise judgement, two separate relative scales of valence and concreteness were constructed as follows: when a word was chosen as most abstract/negative, it receives a score of −1. When a word is chosen as most concrete/positive, it receives a score of +1. These scores are averaged for each word across the number of presentations (*n* = 16), determining the position of every word on the scale. The relative scales obtained from the experiment were compared with the original valence and concreteness ratings using the Kendall rank correlation, as only the relative position of every word matters and the exact value of the score is not necessarily meaningful. The strength of the Kendall correlation in individual patients was compared with HCs using a modified *t*-test implemented in the Singlims programme.^43^

Next, the relative scales for valence and concreteness were combined into a 2D space, which was then mapped onto the space created using the stimulus ratings and after applying Procrustes analysis (vegan package for R 4.2.0). Procrustes analysis determines a transformation (scaling and rotation) that allows for the optimal mapping of one configuration onto a target configuration (i.e. the stimulus ratings). It is particularly useful in the comparison of ordination results. The best solution is determined by minimising an error term. Here, we compared the total error in individual patients to mean error in the HCs using Singlims. When the error in a patient was significantly higher than can be expected in a healthy population, stimulus-specific error was further investigated using one-way ANOVA to determine whether significant differences between stimulus groups exist.

## Results

The individual neuropsychological test scores for each of the patient participants are presented in Table 3. For the CDR, Pyramids and Palm Trees test (PPT), Psycholinguistic Assessment of Language Processing in Aphasia (PALPA) and the Werkwoorden-en Zinnentest test, pathological scores were identified through comparison with published norms. For the Auditory Verbal Learning Test (AVLT), Boston Naming Test (BNT) and the Animal Verbal Fluency (AVF) test, pathological scores were identified by means of a linear regression model derived from a cognitively intact control group (*n* = 180) with test score as dependent variable and age, education and sex as predictors. Patients scores > 1.5 * prediction interval were identified as pathological.

**Table 3:** Neuropsychological tests scores for each of the patient participants

	*K01*	*K02*	*K03*	*K04*	*K05*	*K06*	*K07*	*K08*	*K09*	*K10*
*Age*	64	58	70	65	76	61	68	72	60	66
*Sex*	F	M	F	F	F	F	M	M	M	F
*Education (years)*	17	14	15	12	12	13	16	17	17	14
*MMSE (/30)*	25	25	**12**	29	29	24	30	28	26	29
*CDR-G*	**0.5**	**0.5**	**1**	0	0	**0.5**	**0.5**	**0.5**	0	**0.5**
*BNT, %*	**5**	**25**	**16.67**	**30**	**45**	**13.33**	93.33	**41.66**	**63.83**	**63.64**
*CPM (/36)*	36	32	29	29	28	33	34	33	33	32
*PPT (/52)*	42	**40**	**37**	45	50	**36**	51	52	48	48
*AVF*	**5**	**7**	**6**	16	**8**	**5**	24	16	24	17
*AVLT* t*otal learning*	**27**	**34**	**7**	**27**	**22**	**28**	41	**26**	39	52
*Delayed recall, %*	0.83	**0.25**	NA	0.71	**0**	**0.17**	**0**	0.67	0.83	0.7
*Delayed* r*ecall (/15)*	11	**9**	NA	**9**	15	12	**9**	**9**	13	11
*PALPA 9 (/80)* s*ingle words*	77	**59**	**70**	80	80	80	80	77	79	77
*Pseudowords*	79	**57**	**56**	78	74	75	80	77	75	**66**
*PALPA 49 (/15)* h*igh imageability*	**10**	13	**9**	15	**12**	**7**	15	**12**	15	13
*Low imageability*	**5**	13	**4**	**11**	**8**	**2**	13	13	13	13
*PALPA 45 (/40)* c*orrect*	**25**	**29**	**34**	38	38	**31**	40	40	39	38
*Semantic errors*	**7**	**7**	**5**	2	2	**5**	0	0	1	2
*Semantic visual*	**4**	**5**	**2**	2	1	1	0	0	0	0
*Distant semantic*	**2**	**1**	**1**	0	0	**3**	0	0	0	0
*Visual*	**3**	**4**	0	0	0	**1**	0	0	0	0
*Unrelated*	**1**	0	0	0	0	0	0	0	0	0
*WEZT* v*erb comprehension (/60)*	**41**	50	**46**	58	53	**46**	60	50	55	54
*Sentence comprehension (/40)*	**31**	39	**27**	38	35	34	40	40	39	40
*Anagrams (/20)*	19	20	**13**	20	20	19	20	20	20	20
*BORB (/32)* h*ard (/32)*	**15**	**16**	**19**	26	26	**21**	26	**19**	25	24
*Easy (/32)*	**26**	**17/31**	30	**27**	28/31	**24**	28/31	**26**	31	28

MMSE, MiniMental State Examination test; CDR-G, clinical dementia rating scale-global score; BNT, Boston naming test; CPM, coloured progressive matrices; PPT, pyramids and palm trees test; AVF, animal verbal fluency; AVLT, auditory verbal learning test; PALPA, psycholinguistic assessment of language processing in aphasia; PALPA 9, (pseudo) word repetition (/80); PALPA 49, semantic association (/15); PALPA 45, word-picture matching (/40); WEZT, Werkwoorden-en Zinnentest: verb and sentence comprehension, sentence anagrams (/60); BORB, Birmingham object recognition battery: object recognition (/32). Bold font indicates pathological scores.

### Data quality

Two HC participants, out of 79, did not perform all three tasks and were excluded from further analysis.

A two-way ANOVA with group as between-subjects factor (two levels: SV versus controls) and task as within-subjects factor (three levels: pairwise valence judgement, pairwise concreteness judgement and triad judgement) and reaction time as outcome yielded a significant main effect of group (*F*(1,83) = 40.8833, *P* < 0.001) and a significant main effect of task (*F*(2,166) = 87.084, *P* < 0.001). No interaction effects were present (*P* > 0.05). According to a Tukey *post hoc* analysis, PPA SV patients were significantly slower (mean = 8.85 s, SD = 0.61) (*P* < 0.0001) than HCs (mean = 8.39 s, SD = 0.42). Reaction times were significantly slower for the concreteness (mean = 8.31 s, SD = 0.32) and for the triad judgement task (mean = 8.90 s, SD = 0.36), than for the valence judgement task (mean = 8.13 s, SD = 0.30) (*P* < 0.001). Reaction times differed significantly between the two groups for each of the three tasks (HC/Concreteness (mean = 8.26, SD = 0.27); SV/Concreteness (mean = 8.68, SD = 0.46) (*P* < 0.001). HC/Valence (mean = 8.07, SD = 0.22); SV/Valence (mean = 8.51, SD = 0.50) (*P* < 0.001). HC/Triad (mean = 8.84, SD = 0.29), SV/Triad (mean = 9.37, SD = 0.51) (*P* < 0.0001)) (see Supplementary Table 2 for further Tukey *post hoc* group/task interaction effects).

Overall, the consistency of responses for repeated pairs or triads within the patient group was satisfactory (Fig. 2). The presence of some variation may result from the inclusion of certain ‘neutral’ words in the stimulus dataset (i.e. words with a valence rating either highly negative or weakly positive). The evaluation of these words as positive or negative might have depended on the specific word they were paired with.

### Triad judgements

**Figure 3:**
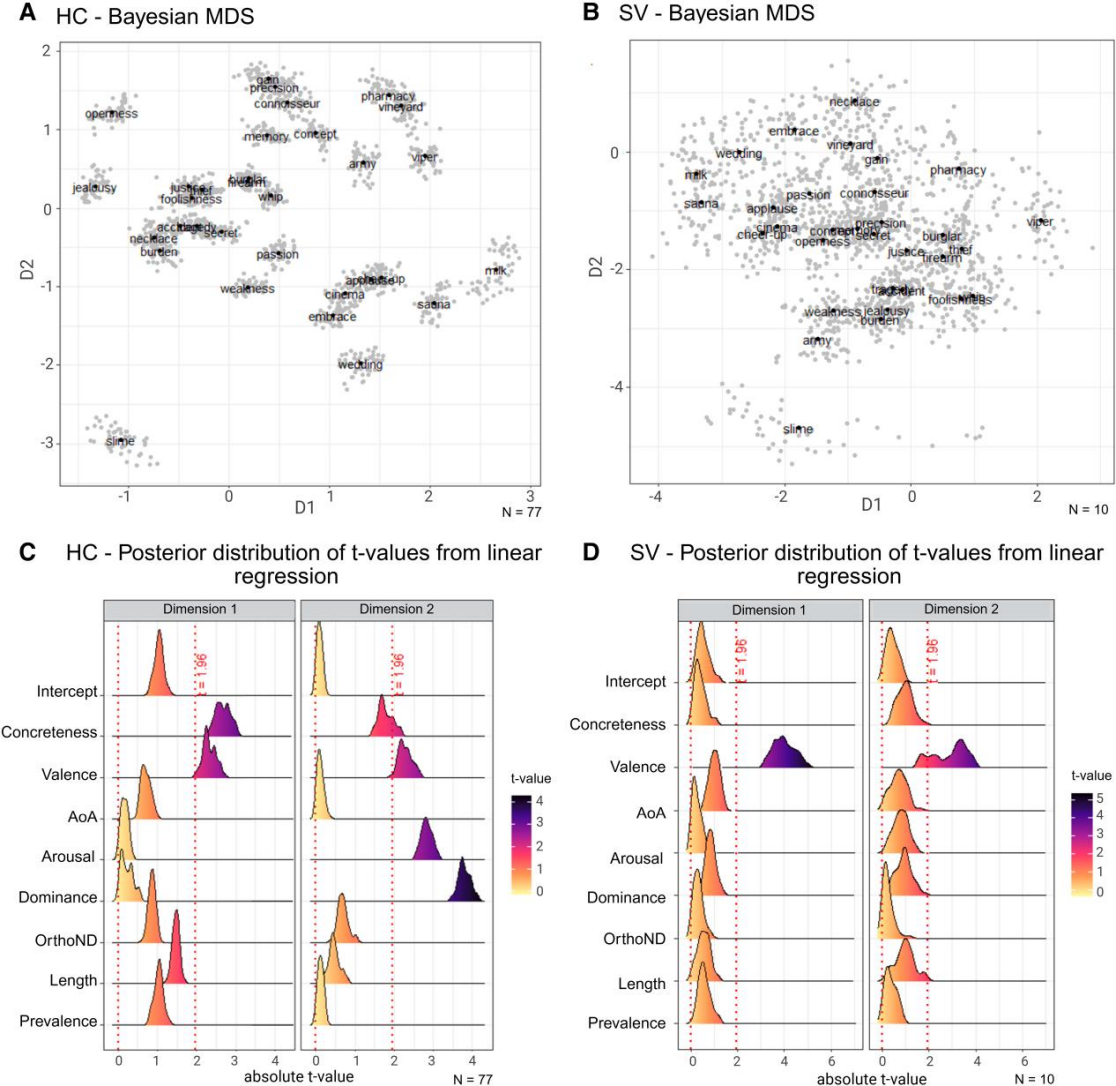
**Bayesian multidimensional scaling (MDS) and posterior distribution of *t*-values from linear regression.** (**A**, **B**) Bayesian MDS based on latent semantic 2D configuration from HC responses and from PPA SV responses, respectively. The posterior mean for the location of each word is shown in black circle (32 points). The 50 posterior samples are shown in grey circles (1600 points). (**C**, **D**) Posterior distribution of *t*-values from a linear regression repeated for each of the 50 posterior samples. The *t*-values > 1.96 (highlighted in red colour) corresponds to *P* < 0.05. D1, dimension 1; D2, dimension 2; AoA, age of acquisition; OrthoND, orthographic neighbourhood density.

The Bayesian MDS is visualized in Fig. 3. A linear regression with posterior mean of the locations as dependent variable and the stimulus ratings as predictors showed that, in HC, the first principal dimension was determined by word concreteness (β = 0.39, *P* = 0.013) and valence (β = 0.34, *P* = 0.028) and the second dimension by word dominance (β = 2.09, *P* = 0.0009) and arousal (β = −1.45, *P* = 0.009) (Fig. 3A and C). This confirmed a successful manipulation of the stimulus material on the dimensions of valence and concreteness. In PPA SV, the semantic network changed to a circumplex configuration (Fig. 3B) with valence as a unique predictor in both dimensions (dimension 1: β = −0.72, *P*  *=* 0.0005; dimension 2: β = 0.46, *P* = 0.006) and no effect of word concreteness (see Supplementary Materials—Fig. 2 for the significant difference between the Bayesian MDS configuration for the HC group and the Bayesian MDS configuration for each SV PPA patient).

A linear regression performed on each of the 50 posterior samples confirmed the role of concreteness, valence, arousal and dominance in predicting HC responses, and the importance of only valence in predicting PPA SV responses (Fig. 3C and D). It is worth noting that dominance, valence and arousal, are three variables characterizing the affective component of emotions.^4-30,44^

**Figure 4:**
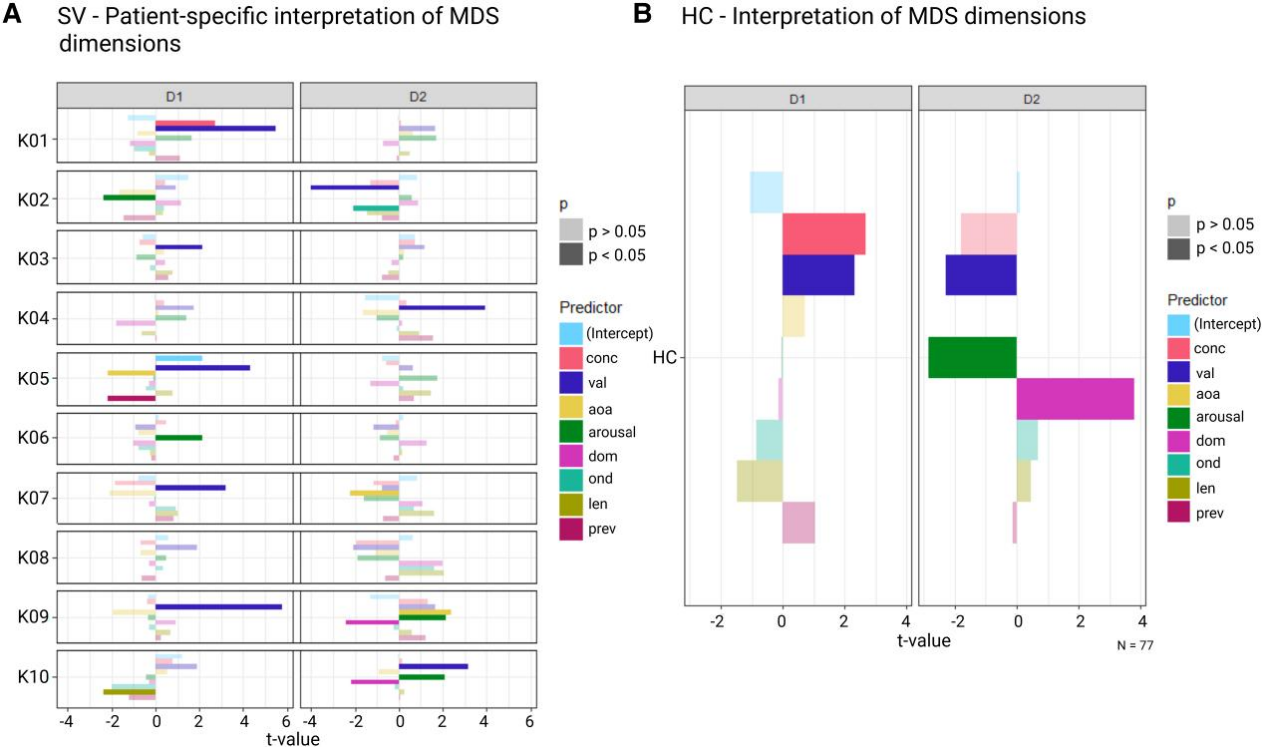
**Interpretation of multidimensional Scaling (MDS) dimensions.** (**A**) Patient-specific determinants of Bayesian MDS dimensions. For each patient and each MDS dimension (D1 and D2), a separate multiple linear regression model was fitted using the following predictors: concreteness, valence, age of acquisition (AoA), arousal, dominance, orthographic neighbourhood density (OND), word length, and prevalence. The plot reports the *t*-values associated with each regression coefficient (i.e. the test statistics). Significance was assessed using the standard *t*-distribution from the linear model (lm) in R (version 4.2.0). This visualization at the individual level is based on a comparable analysis to that used for Fig. 3D at the group level. (**B**) Interpretation of Bayesian MDS dimensions for HC pooled. The same multiple regression model was applied at group level for HC. This group-based analysis visualizes the same outcome as Fig. 3C in a different way. Transparency represents significance: opaque bars correspond to *P* < 0.05, while transparent bars indicate *P* > 0.05. conc, concreteness; val, valence; aoa, age of acquisition; dom, dominance; ond, orthographic neighbourhood density; len, length in letters; prev, prevalence; D1, dimension 1; D2, dimension 2; HC, healthy controls.

Bayesian MDS dimensions were further investigated at patient-specific levels by computing a linear regression for each patient (Fig. 4). Valence was a significant (*P* < 0.05) predictor in 8 out of 10 SV patients, followed by arousal (which was significant in 4 out of 10 PPA SV patients (K02, K06, K09 and K10)) and age of acquisition (significant in 3 out of 10 PPA SV patients). Concreteness was a significant predictor (*P* = 0.01) in only one patient (K01), together with valence (*P* < 0.0001). In only one case (K07) was concreteness numerically stronger as predictor than valence. In retrospect, this case was peculiar since the patient in question had a BNT score of 56/60, which is highly unusual for PPA SV. Clinically, in retrospect, the main complaints of this case were amnestic despite the bilateral anterior temporal hypometabolism on FDG PET. Accordingly, analysis was repeated by excluding this case. Results remained essentially the same (Supplementary Fig. 3).

Finally, spatial randomness was computed over all patients pooled, over all HC pooled and for each patient separately (Supplementary Fig. 4). An analysis was computed for each of the 50 posterior samples. Compared with HC (mean = 0.75, SD = 0.03), spatial randomness over all PPA SV patients pooled showed a distribution moving towards *R* = 1 (mean = 0.81, SD = 0.07), which implies a degraded semantic clustering (Supplementary Fig. 4A). The 50 posterior samples distribution for the PPA SV was significantly different from the distribution for the HC (Kolmogorov–Smirnov test, *P* < 0.0001). The PPA SV pattern was confirmed by a patient-specific spatial randomness effect, where half of the patients had a mean slightly higher than 1 - K01 (mean 1.02, SD = 0.10), K05 (mean = 1.05, SD = 0.09), K07 (mean = 1.00, SD = 0.09), K08 (mean = 1.07, SD = 0.09), K09 (mean = 1.01, SD = 0.08)—and the remaining half slightly <1—K02 (mean = 0.99, SD = 0.09), K03 (mean = 0.99, SD = 0.08), K04 (mean = 0.97, SD = 0.11), K06 (mean = 0.89, SD = 0.11) and K10 (mean = 0.96, SD = 0.09) (Supplementary Fig. 4B). For completeness, the analysis was also carried out over every HC separately. Distributions are reported in Supplementary Fig. 5.

### Pairwise judgements

**Figure 5:**
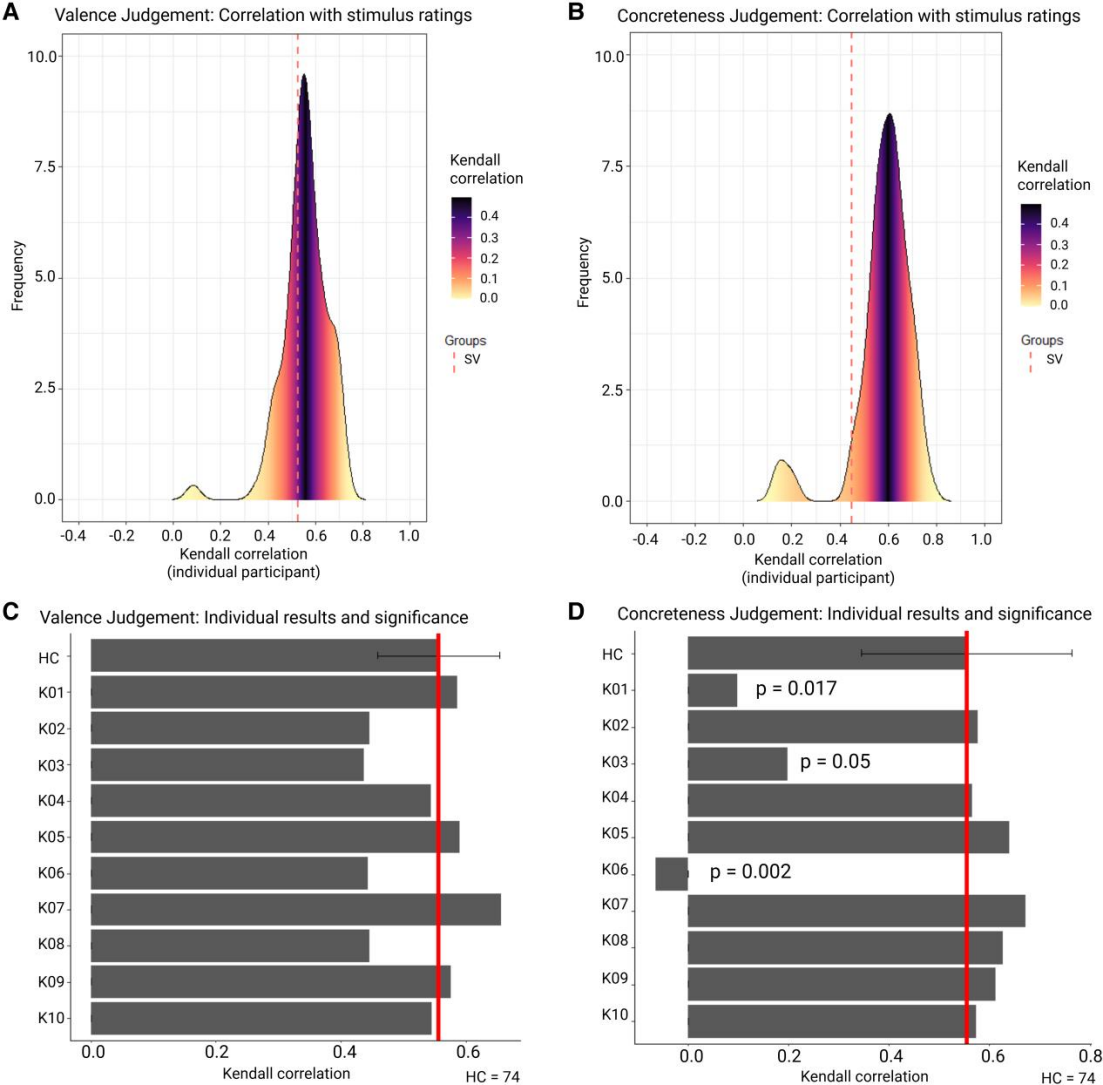
**Kendall correlation with literature-based stimulus ratings and stimulus ratings based on the participant’s responses.** (**A**, **B**) Distribution of the Kendall correlation coefficients between the literature-based valence (**A**) or concreteness (**B**) stimulus ratings and the stimulus ratings based on individual participant’s responses. The red dashed line indicates the group-averaged Kendall correlation for PPA SV. The line is drawn against the background of the distribution where each point corresponds to the Kendall correlation computed for an individual HC participant (HC = 74). (**C**, **D**) Barplot of the Kendall correlations for valence (**C**) and concreteness (**D**) in the group of the HCs (first row, mean, and standard deviation) and for each of the SV cases (row 2–11). For visualization purposes, the red solid lines indicate the mean value in HCs. *P*-values refer to the significance of the difference in the correlation obtained in an individual patient compared with the correlations in the HC group. Significance of the difference was obtained by using a modified *t*-test implemented in the Singlims programme. Only *P*-values < 0.05 are reported. HC, healthy control (*N* = 74); SV, semantic variant.

For the valence pairwise judgement, in HC the mean Kendall correlation between the obtained relative scores and valence stimulus ratings was 0.55. Similarly, in PPA SV patients, the mean Kendall correlation between relative scores and valence stimulus ratings was 0.53 (Fig. 5A). Compared with HC, none of the PPA SV patients had a significantly higher correlational value (Fig. 5C). In brief, we did not find valence judgement to be significantly affected in SV for the word set used.

For the concreteness pairwise judgement, in HCs the mean Kendall correlation between the obtained relative scores and concreteness stimulus ratings was 0.55, while the mean correlation between PPA SV patients and concreteness ratings was 0.45 (Fig. 5B). The two mean Kendall correlations were not significantly different (*P* = 0.3). Compared with HC, three SV patients showed a significantly lower Kendall correlational value (Fig. 5D).

**Figure 6:**
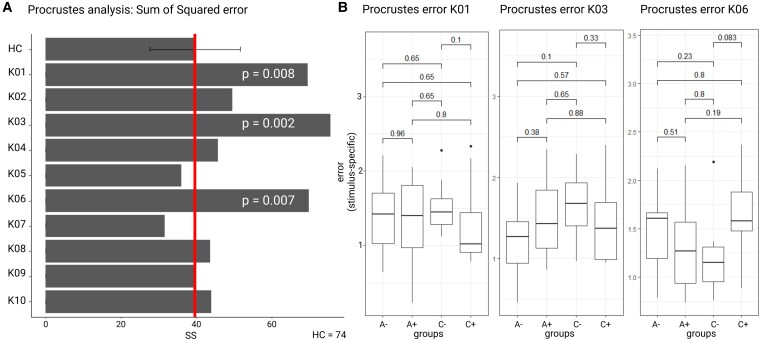
**Procrustes analysis.** (**A**) Mean and SD of sum of squared error (SS) over HC (*N* = 74) and SV patient-specific sum of squared error. For visualization purposes, the red solid lines indicate the mean value in HCs. *P*-values refer to the significance of the difference between total sum of squared error in HC and patient-specific sum of squared error. Significance of the difference was obtained by using a modified *t*-test implemented in the Singlims programme. Only *P*-values < 0.05 are reported. (**B**) For each SV case (K01, K03 and K06), bars show stimulus-specific errors for each of the four stimulus groups. Significance of the difference between stimulus groups was assessed by using the stat_compare_means () function from the ggpubr package in R (version 4.2.0). A−, abstract negative; A+, abstract positive; C−, concrete negative; C+, concrete positive.

Finally, procrustes analysis revealed that three PPA SV patients showed significantly higher procrustes sum of squared (SS) error than HC (Fig. 6A). A higher SS value indicates a worse match with the target configuration. In all three PPA SV patients, stimulus-specific errors were not linked (*P* > 0.05) to a specific stimulus group (Fig. 6B).

Three cases (K01, K03 and K06) showed the most extreme pattern, with significant impairment of concreteness judgement. These three cases also showed the lowest BNT scores of the entire group.

## Discussion

Using state-of-the-art semantic mapping approaches at the group and at the individual level, the current study demonstrates the distortion of the semantic space in PPA SV: the role of concreteness in the organization of the semantic network is reduced or obliterated while the effect of valence is remarkably preserved. The group data and the individual-level analysis were highly consistent, where the case with the highest BNT score (K07) did not show the abnormal pattern and the three cases with the lowest BNT scores (K01, K03 and K06) showed the distortion to the highest degree. The current findings provide evidence of a distortion pattern according to which the semantic space degrades in PPA SV. The data raise clinically relevant questions with regards to how word entries and word meaning are affected by the progressive neurodegeneration in PPA SV, and have also more fundamental implications for models of word meaning.

Previous studies focused on which word categories were more likely affected in the early stages of PPA and revealed the concreteness reversal effect.^6,45^ At a group level, abstract words, e.g. denoting emotion, are better preserved in PPA SV than concrete words, with the exception of social relation words.^7^ However, the comparison in the current study is not so much between different classes of words. The analysis pertains to how the semantic space is structured in PPA SV. The important distinction with the dissociation between concrete and abstract words is that across words, an aspect of word meaning is preserved (valence) while other aspects of word meaning are lost (concreteness). The lexical entry is not lost at this disease stage and the valence associated with the lexical entry is relatively preserved. However, the concrete features that are bound together with this lexical entry are affected. A loss of the concreteness dimension as organizing principle of the semantic network is distinct from the previously reported loss of concrete words (concreteness reversal): Abstract words also vary in their degree of concreteness/abstractness. A loss of the concreteness dimension also implies that the content of abstract words may be altered and the meaningful content related to more concrete aspects is diminished across the different word classes, both abstract and concrete.

In PPA, spontaneous speech tends towards a higher proportion of abstract words.^5^ Intuitively, one could attribute this ‘*ex vacuo*’ to the loss of concrete words. Abstract words have higher ratings of positive or negative valence.^46^ Our findings lead to the hypothesis that the preponderance of abstract words is as a reflection of a valence-dominated semantic space rather than a logical consequence of the reduction of concrete word use.

The current findings have clinical implications. Clinically, patients with PPA SV are often tested based on confrontation naming, which is based on concrete items or actions, on verbal fluency for animals or other concrete categories. Word-picture matching and object decision are equally based on concrete features. Also the Pyramids and Palm Trees test and its variants have been traditionally based on concrete items, as they necessitate pictorial analogues for each of the words. These tasks have all in common that they probe concrete, imageable entities and hence provide a biased view of how the semantic space is organized in an individual. The semantic space encompasses other aspects than the sensory features and the concrete objects that are commonly probed. Therefore, based on the current findings, we put forward that we underestimate the comprehension and word knowledge of patients with PPA SV by focusing on concrete words and pictures of their referents. Hence, the clinical bias towards judging performance based on concrete items may underestimate the inner mental life of patients with PPA SV. We speculate that the semantic space is in a valence-dominated disequilibrium rather than carved based on a balanced representation of valence and concreteness.

Episodic memory is more emotional and personal, autonoetic than semantic memory.^47^ Unlike semantic memory, core characteristics of autobiographical/episodic memory include affective internal contextual details, which allow visual and emotional re-experiencing of an event. The relative preservation of valence in PPA SV, a disease characterized by degradation of semantic memory, is in line with this model.

Different competing theories exist regarding the representation of word meaning. There is a consensus that concrete, sensory features are represented in the unimodal association cortices that also process these features in the presence of the object.^1^ A key discussion point is whether additional regions are involved in binding these features together into the concept to which the word refers. Based on the hub-and-spoke theory,^3^ the anterior temporal cortex fulfils this binding role, as a hub, with the sensory unimodal cortices serving as spokes. In its strong formulation, the neural network for semantic memory requires a single convergence zone or hub that supports interactive activation of representations in all modalities, for all semantic categories.^1^ Notwithstanding, it is noteworthy that the concrete formulation of the hub-and-spoke theory almost exclusively pertains to concrete entities and only refers to a very limited degree to abstract concepts. The current data may provide for a reason. The anterior temporal lobe may be particularly relevant for binding sensory features that define (part of) the meaning of concrete words. More abstract characteristics of words may rely on different regions. Hence, the anterior temporal cortex does not serve as a hub in the sense of binding all components of word meaning into an amodal concept, but more in the sense of binding the concrete, sensory features together and liaise it with the lexical entry. Based on this hypothesis, in the intact brain the binding of valence together with the node’s concrete features may rely on different regions than the anterior temporal cortex or be represented in a more distributed manner. Category-dependent differences in the neuroanatomical organization of convergence within the anterior temporal cortex are a key component of the graded hub-and-spoke hypothesis, a more nuanced and differentiated version of the originally formulated hub-and-spoke theory.^3^ Based on the heteromodal theory, possible regions that may play a role outside the anterior temporal cortex are the superior temporal gyrus and posterior third of the temporal gyrus or the angular gyrus.^2^ Macoir *et al*.,^18^ examined the effect of valence on written word processing in eight PPA SV cases and found that the processing of word valence was more closely linked to performance on lexical decision than with associative-semantic processing. This is in line with the hypothesis that the lexical entry and its valence features can be preserved while the semantic associations may be degraded. Neurophysiologically, the special status of word valence is also evident from the early posterior negativity, an effect of word valence on ERP that occurs 200 ms after written word onset,^20^ i.e. much earlier than what is required for semantic effects.

As a limitation, the current study design is cross-sectional. Longitudinal measures may provide further insight into the effect of disease progression on the organization of the individual’s semantic space. Second, while the eligibility criteria required sufficient cooperation and comprehension to be able to obtain reliable results, there is still a variation in disease severity. The worst affected cases also showed the strongest distortion of the semantic space, indicating the clinical relevance of our findings. Furthermore, the sample did not allow for subgroup analysis comparing cases with left, right, or bilateral anterior temporal degeneration to discern the relative contribution of the two hemispheres to word valence processing.

To conclude, in PPA SV, valence is relatively preserved as an organizational principle of the semantic space, in contrast with concreteness.

## Supplementary Material

fcaf281_Supplementary_Data

## Data Availability

Data will be made available via RDR, the KU Leuven's institutional research data repository for the publication of research data.

## Supplementary material

Supplementary material will be available at *Brain Communications*  online.
