# Supplementary material for: Valence as principal dimension of the semantic space in primary progressive aphasia semantic variant
Source: Brain Commun. 2025 Jul 23;7(4):fcaf281. doi: 10.1093/braincomms/fcaf281 (PMC12341893; doi:10.1093/braincomms/fcaf281)
Supplement: fcaf281_Supplementary_Data [file fcaf281_supplementary_data.docx]

## Supplementary materials


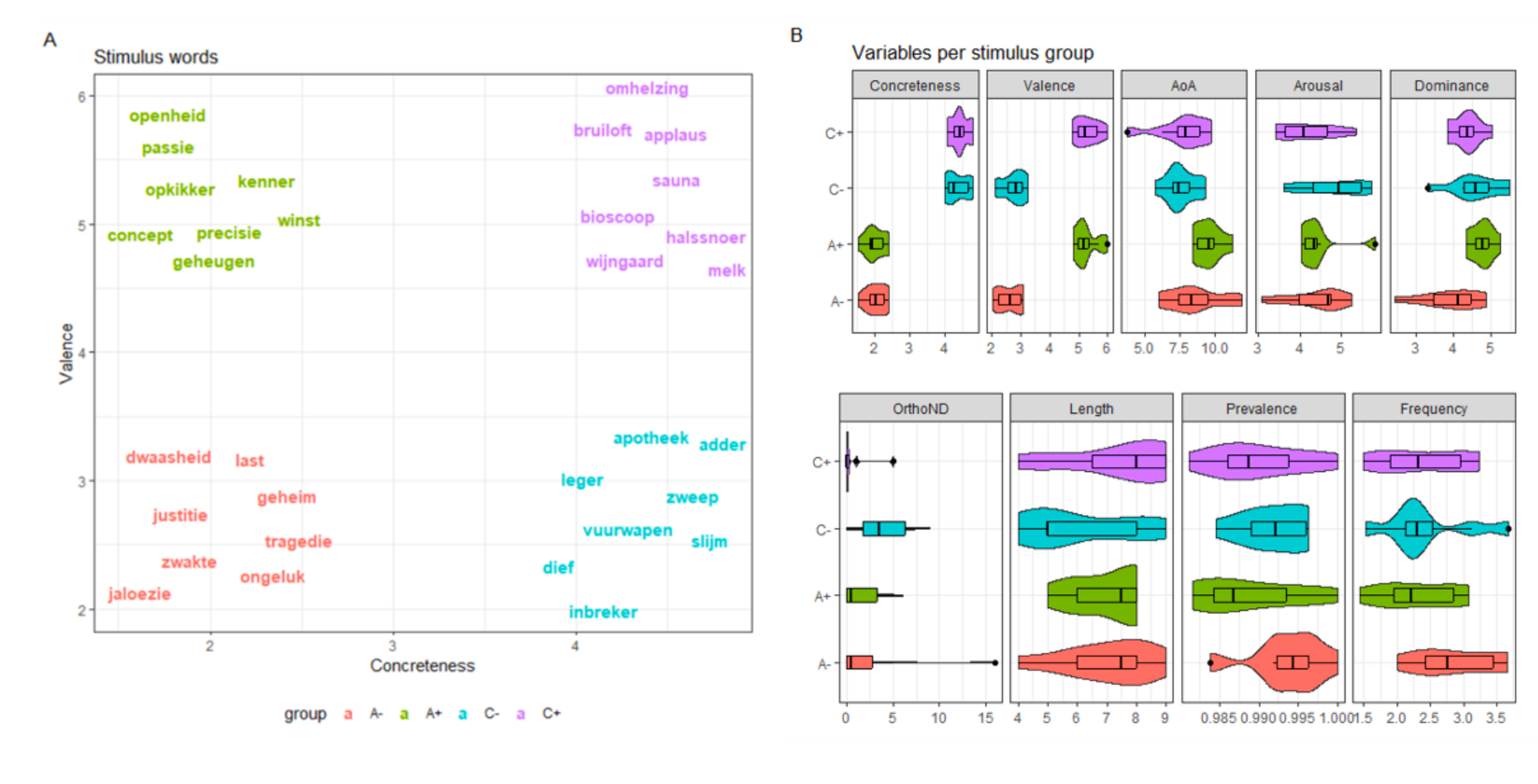


**Supplementary Fig 1:** **A.** Spatial distribution of individual words in a two-dimensional stimulus space defined by their concreteness and valence ratings. **B.** For each of the four stimulus classes from the factorial design, violin plots show the distribution of word-level features (as indicated in each plot title). Each plot displays the median and the interquartile range (box), based on all words within the respective stimulus group. *Abbreviations*: A-: Abstract negative. A+: Abstract positive. C-: Concrete negative. C+: Concrete positive.

# Triad Judgments

In order to address the relation between the Bayesian MDS configuration of the HC group with each patient-specific Bayesian MDS configuration, a procrustes analysis was computed between each subject-specific (HC and SV patient) two-dimensional MDS configuration and a two-dimensional configuration for the Small World of Words (SWOW) dataset (i.e., target configuration). Significance of the difference between total error in individual patients and mean error in the healthy controls was performed by using Singlims. Results revealed that individual errors of 6 out of 10 SV PPA patients were significantly different from mean error in HC.

**
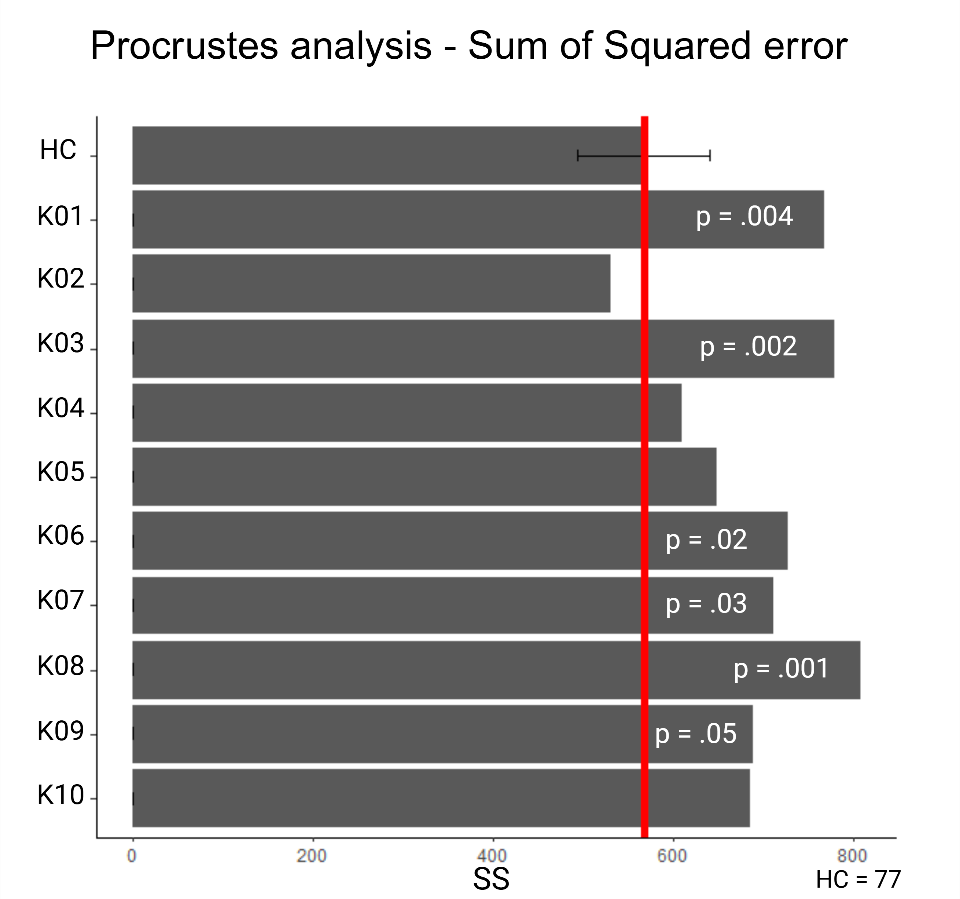
Supplementary Figure 2**: Procrustes analysis. Mean and standard deviation of sum of squared error (SS) over Healthy Controls (HC) (N = 77) and SV patient-specific sum of squared error. For visualization purposes, the red solid lines indicate the mean value in healthy controls. P-values refer to the significance of the difference between total sum of squared error in HC patient-specific sum of squared error. Significance of the difference was obtained by using a modified t-test implemented in the Singlims program. Only p-values < .05 are reported


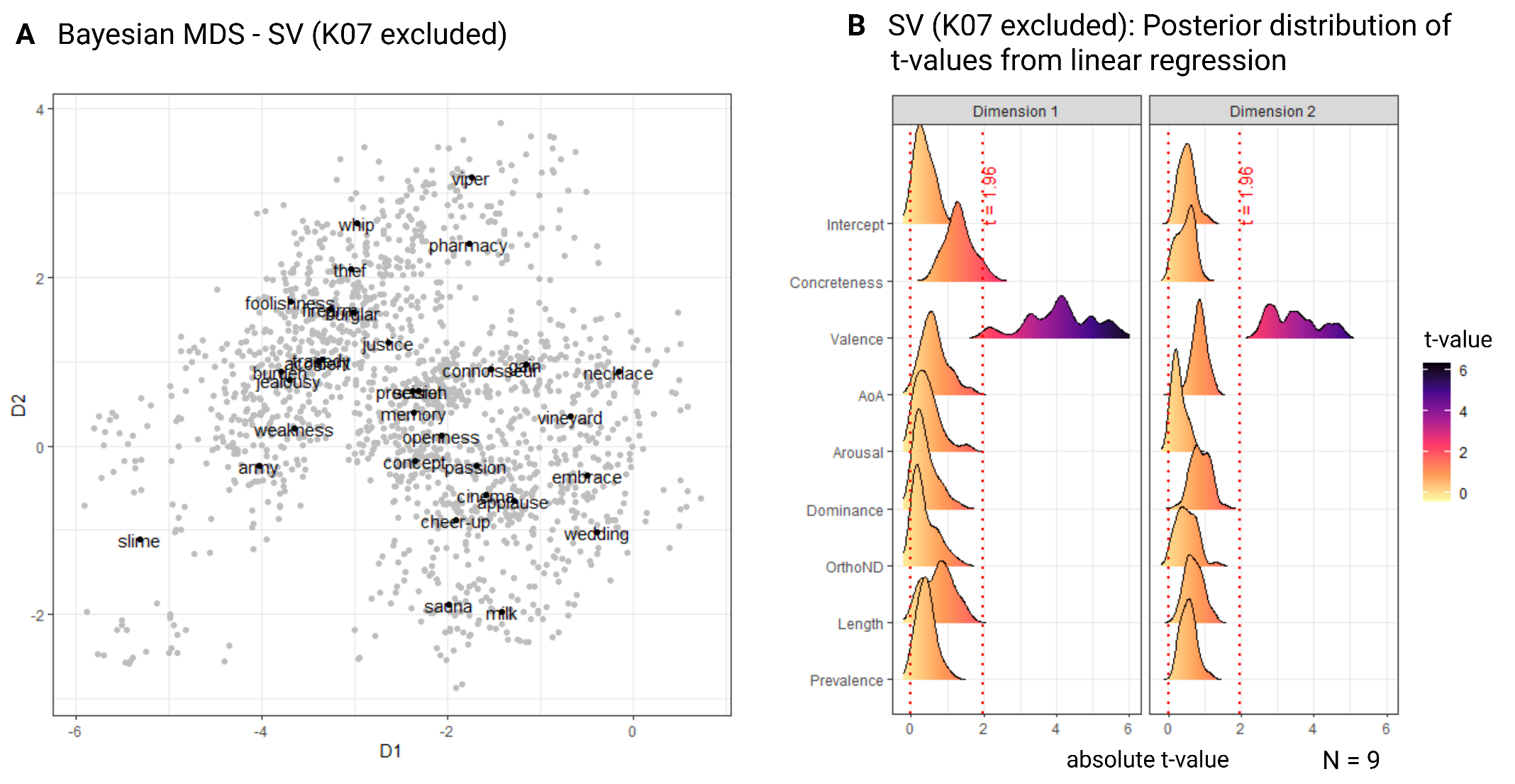


**Supplementary Figure 3:** **(A)** Bayesian Multidimensional scaling (MDS) based on latent semantic two-dimensional configuration from PPA SV responses of 9 out of 10 subjects (K07 excluded). The posterior mean for the location of each word is shown in black circle (32 points). The 50 posterior samples are shown in grey circles (1600 points). **(B)** Posterior distribution of t-values from a linear regression repeated for each of the 50 posterior samples. T-values > 1.96 (highlighted in color) corresponds to p < .05. *Abbreviations*: SV = Semantic variant;


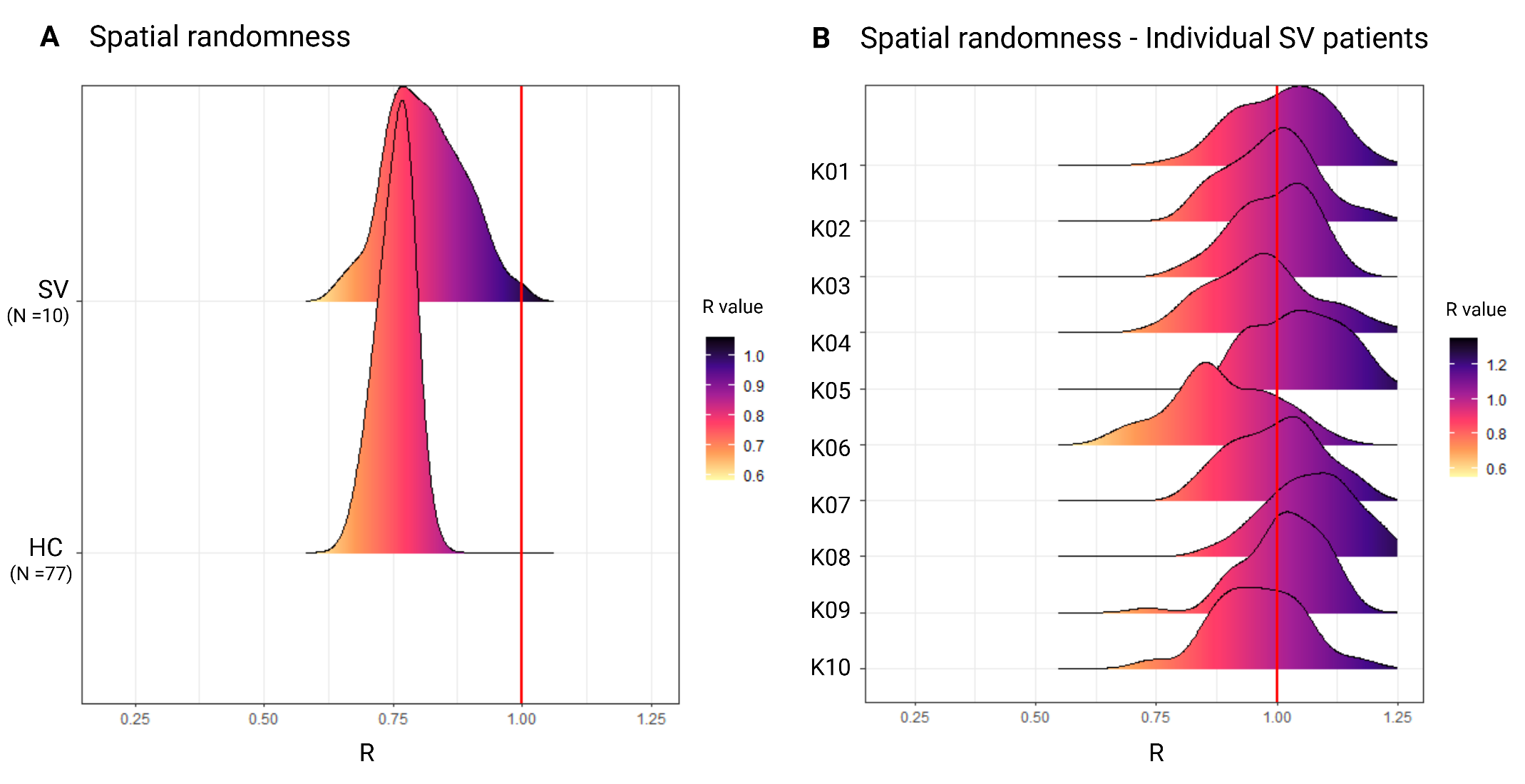


**Supplementary Figure 4: (A)** Spatial randomness over PPA SV patients pooled (upper row) and HC pooled (lower row). For each population, a distribution of R values for the 50 posterior samples is shown. The model is based on all trials from all patients (i.e., 1790 trials) and likewise for the controls. **(B)** Distribution of R values for the 50 posterior samples for each PPA SV patient separately (179 trials per case). Abbreviations: HC = Healthy controls; SV = Semantic variant.


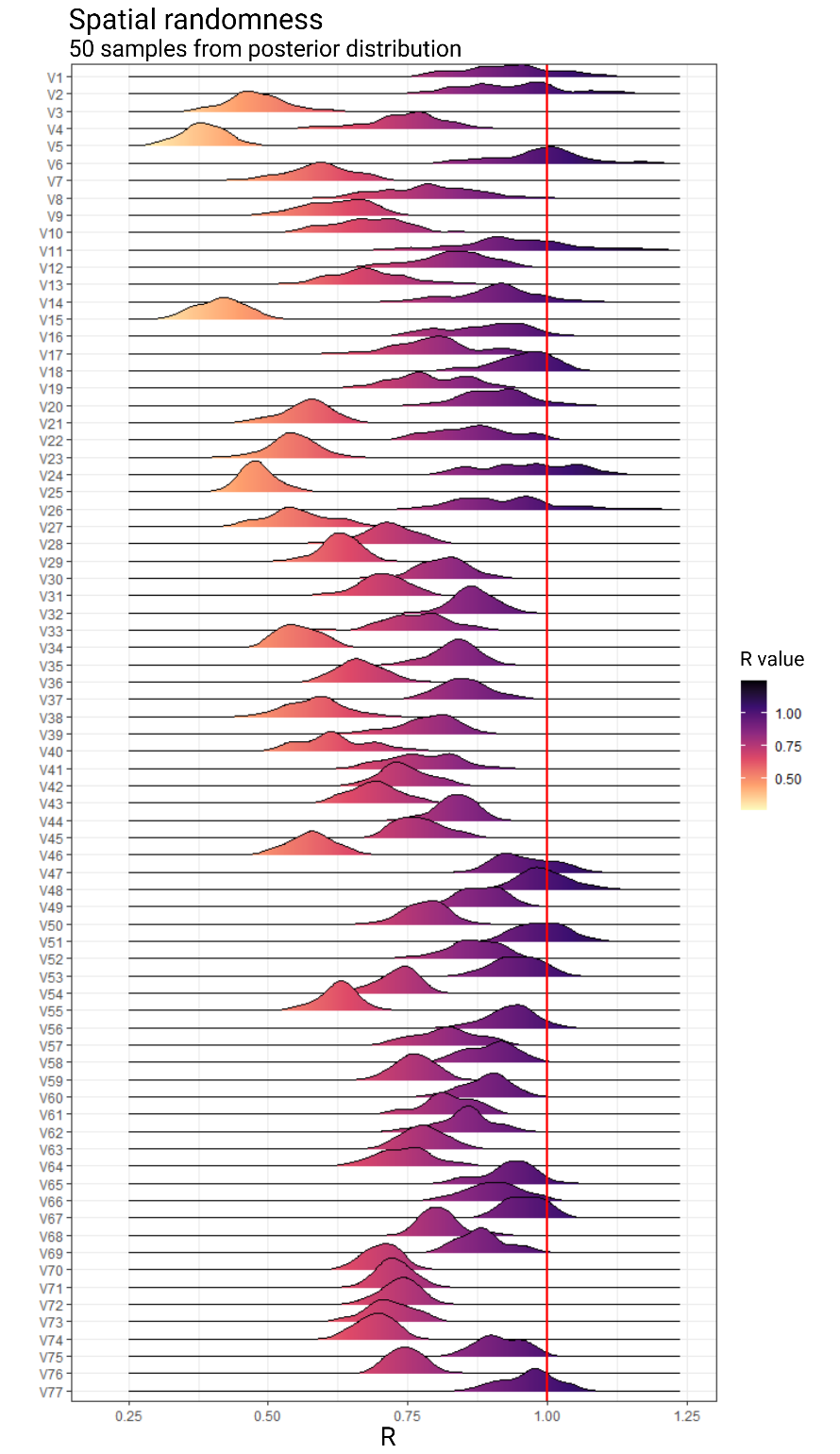


**Supplementary Figure 5:** Distribution of spatial randomness parameter R (Lee et al., 2016) for the 50 posterior samples for each of the 77 individual HC. An R-value < 1 represents increasing levels of clusters, while R > 1 represents a shift towards a regular grid, which implies a spatially random MDS configuration lacking of semantic clustering.

| WORD | WORD EN | Conc | Val | AoA | Arou | Dom | Orthond | Length | Prev | Freq | Group |
| --- | --- | --- | --- | --- | --- | --- | --- | --- | --- | --- | --- |
| winst | profit | 2,40 | 5,15 | 8,58 | 4,51 | 4,72 | 6 | 5 | 0,98540146 | 2,98 | A+ |
| precisie | precision | 2,20 | 5,05 | 10,40 | 4,05 | 4,83 | 0 | 8 | 0,988142292 | 2,01 | A+ |
| geheugen | memory | 1,93 | 4,83 | 8,40 | 4,38 | 4,63 | 0 | 8 | 1 | 3,08 | A+ |
| concept | concept | 1,53 | 4,82 | 11,30 | 4,10 | 4,42 | 1 | 7 | 0,985018727 | 2,41 | A+ |
| passie | passion | 1,86 | 5,72 | 9,58 | 5,81 | 5,24 | 3 | 6 | 0,992592593 | 2,81 | A+ |
| opkikker | pick-me-up | 1,93 | 5,17 | 9,72 | 4,13 | 4,33 | 0 | 8 | 0,981884058 | 1,45 | A+ |
| kenner | expert | 2,40 | 5,23 | 8,83 | 4,35 | 4,95 | 4 | 6 | 0,996 | 2,02 | A+ |
| openheid | openness | 1,86 | 5,97 | 9,61 | 4,34 | 4,93 | 0 | 8 | 0,981549815 | 1,74 | A+ |
| dwaasheid | foolishness | 1,87 | 3,09 | 9,00 | 4,08 | 3,41 | 0 | 9 | 0,992395437 | 2,00 | A- |
| zwakte | weakness | 1,80 | 2,27 | 8,40 | 3,08 | 2,46 | 5 | 6 | 0,996282528 | 2,41 | A- |
| ongeluk | accident | 2,26 | 2,16 | 6,02 | 4,66 | 4,35 | 0 | 7 | 0,996323529 | 3,65 | A- |
| geheim | secret | 2,33 | 2,99 | 6,46 | 4,75 | 4,09 | 2 | 6 | 0,991869919 | 3,65 | A- |
| jaloezie | jealousy | 1,53 | 2,02 | 7,77 | 5,25 | 4,81 | 0 | 8 | 1 | 2,42 | A- |
| justitie | justice | 1,93 | 2,85 | 10,90 | 4,75 | 4,86 | 0 | 8 | 0,983870968 | 2,89 | A- |
| last | burden | 2,13 | 3,06 | 8,20 | 4,72 | 4,14 | 16 | 4 | 0,992565056 | 3,37 | A- |
| tragedie | tragedy | 2,40 | 2,43 | 11,90 | 3,67 | 3,49 | 1 | 8 | 0,996197719 | 2,62 | A- |
| omhelzing | embrace | 4,13 | 5,97 | 7,77 | 4,64 | 4,61 | 0 | 9 | 0,985611511 | 1,87 | C+ |
| bioscoop | cinema | 4,33 | 5,18 | 8,05 | 4,38 | 4,23 | 0 | 8 | 0,987603306 | 2,67 | C+ |
| halssnoer | necklace | 4,73 | 5,02 | 8,72 | 3,50 | 4,30 | 0 | 9 | 0,981132075 | 1,49 | C+ |
| melk | milk | 4,80 | 4,76 | 3,69 | 3,70 | 3,84 | 5 | 4 | 0,993031359 | 3,24 | C+ |
| sauna | sauna | 4,46 | 5,24 | 9,59 | 3,82 | 4,41 | 1 | 5 | 0,989761092 | 1,98 | C+ |
| applaus | applause | 4,46 | 5,60 | 6,24 | 5,34 | 5,02 | 0 | 7 | 0,996212121 | 2,88 | C+ |
| bruiloft | wedding | 4,06 | 5,63 | 7,71 | 4,70 | 4,51 | 0 | 8 | 1 | 3,20 | C+ |
| wijngaard | vineyard | 4,40 | 4,83 | 9,72 | 3,43 | 3,85 | 0 | 9 | 0,986111111 | 1,91 | C+ |
| apotheek | pharmacy | 4,33 | 3,24 | 7,50 | 3,98 | 4,37 | 0 | 8 | 0,996015936 | 2,29 | C- |
| slijm | slime | 4,64 | 2,65 | 6,84 | 3,62 | 3,31 | 4 | 5 | 0,991525424 | 2,13 | C- |
| inbreker | burglar | 4,06 | 2,10 | 7,25 | 5,51 | 4,78 | 2 | 8 | 0,996389892 | 2,30 | C- |
| dief | thief | 4,00 | 2,23 | 5,69 | 5,43 | 4,78 | 7 | 4 | 0,992882562 | 3,12 | C- |
| adder | adder | 4,80 | 3,18 | 8,80 | 4,43 | 4,25 | 3 | 5 | 0,988636364 | 1,53 | C- |
| leger | army | 4,13 | 2,91 | 7,04 | 5,71 | 5,49 | 9 | 5 | 0,989247312 | 3,67 | C- |
| vuurwapen | firearm | 4,20 | 2,74 | 9,30 | 5,35 | 5,34 | 1 | 9 | 0,996138996 | 2,12 | C- |
| zweep | whip | 4,73 | 2,99 | 7,96 | 4,52 | 4,29 | 6 | 5 | 0,984496124 | 2,33 | C- |

**Supplementary Table 1:** Word specific norms. *Abbreviations:* Word EN: English translation; Conc: concreteness; Val: valence; AOA: age of acquisition; Arou: arousal; Dom: dominance; OrthoND: orthographic neighborhood density; PREV: prevalence; Freq: frequency.

| Tukey Post-hoc analysis – Interaction effects | | | | |
| --- | --- | --- | --- | --- |
| Group1/Task | Group2/Task | Mean and SD (Group1/Task) | Mean and SD (Group 2/Task) | p-value |
| HC / Concreteness | SV / Concreteness | Mean = 8.26s- SD= 0.27 | Mean = 8.68s – SD = 0.46 | p < .001 |
| HC / Concreteness | HC / Valence | Mean = 8.26s - SD= 0.27 | Mean = 8.07s – SD = 0.22 | p <.01 |
| HC / Concreteness | SV/ Valence | Mean = 8.26s - SD= 0.27 | Mean = 8.51s – SD = 0.50 | N.S |
| HC / Concreteness | HC / Triads | Mean = 8.26s - SD= 0.27 | Mean = 8.84s – SD = 0.29 | p <.0001 |
| HC / Concreteness | SV / Triads | Mean = 8.26s - SD= 0.27 | Mean = 9.37s – SD = 0.51 | p <.0001 |
| SV / Concreteness | HC / Valence | Mean = 8.68s – SD = 0.46 | Mean = 8.07s – SD = 0.22 | p <.0001 |
| SV / Concreteness | SV/ Valence | Mean = 8.68s – SD = 0.46 | Mean = 8.51s – SD = 0.50 | N.S. |
| SV / Concreteness | HC / Triads | Mean = 8.68s – SD = 0.46 | Mean = 8.84s – SD = 0.29 | N.S. |
| SV / Concreteness | SV / Triads | Mean = 8.68s – SD = 0.46 | Mean = 9.37s – SD = 0.51 | p <.0001 |
| HC / Valence | SV/ Valence | Mean = 8.07s – SD = 0.22 | Mean = 8.51s – SD = 0.50 | p <.001 |
| HC / Valence | HC / Triads | Mean = 8.07s – SD = 0.22 | Mean = 8.84s – SD = 0.29 | p < .0001 |
| HC / Valence | SV / Triads | Mean = 8.07s – SD = 0.22 | Mean = 9.37s – SD = 0.51 | p < .0001 |
| SV/ Valence | HC / Triads | Mean = 8.51s – SD = 0.50 | Mean = 8.84s – SD = 0.29 | p <.05 |
| SV/ Valence | SV / Triads | Mean = 8.51s – SD = 0.50 | Mean = 9.37s – SD = 0.51 | p < .0001 |
| HC / Triads | SV / Triads | Mean = 8.84s – SD = 0.29 | Mean = 9.37s – SD = 0.51 | p < .0001 |

**Supplementary Table 2:** Tukey post-hoc test for the two-way ANOVA. *Abbreviations:* N.S.: Not significant; SD: Standard Deviation; HC: Healthy control; SV: Semantic variant.

## Supplementary reference

Lee MD, Abramyan M, Shankle WR. New methods, measures, and models for analyzing memory impairment using triadic comparisons. *Behav Res Methods*. 2016;48(4):1492-1507. doi:10.3758/s13428-015-0662-4
